# Esophageal cancer treatment costs by phase of care and treatment modality, 2000‐2013

**DOI:** 10.1002/cam4.2451

**Published:** 2019-07-26

**Authors:** Angela C. Tramontano, Yufan Chen, Tina R. Watson, Andrew Eckel, Chin Hur, Chung Yin Kong

**Affiliations:** ^1^ Institute for Technology Assessment Massachusetts General Hospital Boston Massachusetts; ^2^ Columbia University Medical Center New York City New York; ^3^ Harvard Medical School Boston Massachusetts

**Keywords:** esophageal cancer, healthcare costs, phase of care, SEER‐Medicare, treatment

## Abstract

**Background:**

Detailed cost estimates are not widely available for esophageal cancer. Our study estimates phase‐specific costs for esophageal cancer by age, year, histology, stage, and treatment for older patients in the United States and compares these costs within stage and treatment modalities.

**Methods:**

We identified 8061 esophageal cancer patients in the Surveillance, Epidemiology, and End Results‐Medicare database for years 1998‐2013. Total, cancer‐attributable, and patient‐liability costs were calculated based on separate phases of care—staging (or surgery), initial, continuing, and terminal. We estimated costs by treatment modality within stage and phase for esophageal adenocarcinoma and squamous cell carcinoma separately. We fit linear regression models using log transformation to determine cost by age and calendar year. All costs are reported in 2018 US dollars.

**Results:**

Overall, mean (95% CI) monthly total cost estimates were high during the staging ($8953 [$8385‐$9485]) and initial phases ($7731 [$7492‐$7970]), decreased over the continuing phase ($2984 [$2814‐$3154]), and increased substantially during the 6‐month terminal phase ($18 150 [$17 211‐$19 089]). This pattern of high staging and initial phase costs, decreasing continuing phase costs, and increasing terminal phase costs was seen in all stages. The highest staging costs were in stages III ($9249, $8025‐$10 474) and II ($9171, $7642‐$10 699). The highest initial phase cost was in stage IV, $9263 ($8758‐49 768), the lowest continuing phase cost was in stage I, $2338 ($2160‐$2517), and the highest terminal phase costs were in stages II ($20 533, $17 772‐$23 293) and III ($20 599, $18 268‐$22 929). The linear regression models showed that cancer‐attributable costs remained stable over the study period and were unaffected by age for most histology, stage, and treatment modality subgroups.

**Conclusions:**

Our estimates demonstrate that esophageal cancer costs can vary widely by histology, stage, and treatment. These cost estimates can be used to guide future resource allocation for esophageal cancer care and research.

## 1 INTRODUCTION

Esophageal cancer incidence in the United States has risen over the last 20 years,^1^ with an estimated 17 650 new cases and 16 080 deaths expected in 2019.^2^ The two most common histological subtypes of esophageal cancer are esophageal adenocarcinoma (EAC) and squamous cell carcinoma (ESC). EAC has surpassed ESC as the main subtype in the US, with the number of cumulative cases projected to double between 2011 and 2030 as compared to the prior 20‐year period.^3^ Although there has been an upward trend in overall survival due to advancements in treatment, the 5‐year survival rate of esophageal cancer in the US remains low, at 19.9%, in part due to the fact that the disease is often diagnosed at later stages.^4^, ^5^ As incidence climbs and most esophageal cancers continue to be diagnosed in the advanced stage, improvements in treatment options and early detection methods are urgently needed.

Currently, curative treatment is possible only for patients whose cancer is locally advanced and resectable via esophagectomy, which can have a high rate of complications, particularly among patients with comorbidities, and leads to a decreased quality‐of‐life.^6^, ^7^, ^8^ Minimally invasive esophagectomy (MIE) and endoscopic therapy are new and promising alternatives to open surgery, associated with lower complication rates, shorter hospital stays post surgery, and improved quality of life.^9^, ^10^, ^11^, ^12^ Efforts to reduce esophageal cancer incidence have also assumed greater urgency. One such intervention, radiofrequency ablation, can eliminate Barret's esophagus and dysphagia, considerably lowering the risk of progression to EAC.^13^, ^14^

As standard practice in esophageal cancer care shifts towards newer prevention strategies and treatment modalities, it becomes necessary to consider the trade‐offs between the costs of these interventions and the potential survival benefits they provide. For accurate analyses, a better understanding of the costs of current esophageal cancer modalities is needed.

Costs of esophageal cancer care have previously been estimated using medical claims data.^15^ However, while phase‐specific cost estimates for esophageal cancer care are available in other countries, such as Canada, no comparable studies have been conducted, to our knowledge, in the US context.^16^ In this study, we calculated esophageal cancer treatment costs using the Surveillance, Epidemiology, and End Results (SEER)‐Medicare database for years 1998‐2013, adjusted to US dollars in 2018. We estimated costs by age, year, histology, diagnosis stage, and treatment strategy for a Medicare population. Using these values, we analyzed trends and comparisons between different treatments and phases of care.

## 2 MATERIALS AND METHODS

### 2.1 Cohort inclusion and exclusion

We used the SEER‐Medicare database to calculate costs of esophageal cancer care. SEER‐Medicare is composed of two linked datasets: the SEER dataset of the National Cancer Institute (which collects demographic and clinical information from 18 registries across the US for persons with cancer, representing approximately 28% of the US population),^17^ and Medicare data (which includes health insurance enrollment information as well as outpatient, inpatient, and physician claims for 97% of the population age 65 and older).^18^ There are four components of Medicare coverage. Hospital, skilled nursing, hospice, and some home health care services are covered under Medicare Part A. 96% of Part A beneficiaries are also enrolled in Medicare Part B, which covers physician and outpatient services. Claims data from Medicare Parts C and D are not included in our analysis. Approximately 95% of the SEER registry population aged 65 and older are linked with the Medicare enrollment file.^18^

We used SEER‐Medicare data from years 1998 to 2013 in the analysis and included patients aged 66 or older who were diagnosed with esophageal cancer between 2000 and 2011. We included patients who were diagnosed with EAC or ESC as their first and only cancer and were continuously enrolled in Medicare Parts A and B coverage from 15 months prior to cancer diagnosis until death or December 31, 2013. We defined histology using International Classification of Diseases for Oncology (ICD‐O‐3) codes (Appendix Table A1). Since Health Maintenance Organizations (HMOs) do not submit detailed claims, we excluded those patients who enrolled in an HMO at any time during this period.

We also excluded patients from the analysis if they received Medicare benefits because of end‐stage renal disease or disability, if the month of cancer diagnosis was unknown, if diagnosis was made at autopsy only, or if the date of death recorded in the Medicare database differed from that recorded in the SEER database by more than 3 months. We defined stage using the sixth edition of the American Joint Committee on Cancer (AJCC) Cancer Staging Manual. For those patients diagnosed prior to 2004, we used the SEER variables for extension of disease and lymph node involvement to create the appropriate AJCC 6th edition stage. Patients who had an unknown stage were excluded from the analysis. Lastly, we excluded patients who had costs for claims with unknown dates and those with any costs post death.

### 2.2 Matched control cohort

Our control subjects were beneficiaries from the random sample of 5% of all Medicare enrollees who were aged 65 years and older, who were continuously enrolled in Medicare Part A and B through the study period, who were not enrolled in an HMO, and who had no cancer diagnosis. We matched this control cohort to esophageal cancer patients on an individual (1:1) level within each phase by 5‐year age group, sex, and SEER registry region (Northeast, South, Midwest, West).^19^

### 2.3 Treatment modalities

For patients diagnosed with stages I‐III esophageal cancer, we defined treatment strategies based on treatment(s) initiated 2 months prior to cancer diagnosis through 6 months after diagnosis. We included the 2 months prior to the cancer diagnosis to account for any treatment given to a symptomatic esophageal cancer patient who had not been diagnosed, and to account for possible errors in dates recorded in the claims. For patients diagnosed with stage IV disease, treatment groups were defined by treatment(s) ever received. We defined best supportive care costs as expenses incurred by patients who were not actively treated with surgery, chemotherapy, or radiation. Patients remained in their respective stage, histology, and treatment group throughout the study. For example, a stage II EAC patient who received only surgery within the defined time remained in the surgery group for all phases. A full list of codes used to define surgery, chemotherapy, and radiation can be found in the Appendix Table A1.

### 2.4 Phases of care

We allocated each patient's costs into four separate phases of care—staging (or surgery), initial, continuing, and terminal, defined in terms of months, where “month” refers to a unit of 30 days, regardless of where it falls on the calendar.^19^, ^20^, ^21^

Patients who did not receive surgical treatment during the defined time had a 1‐month staging phase beginning on the date of diagnosis, during which the cancer stage was determined. While in practice it can take longer to determine the disease stage, here we define the phase length based on clinical practice at our institution. To isolate the cost of surgery from that of postoperative care, patients who received qualifying surgery (including local endoscopic therapy) were considered to have a 1‐month surgery phase, beginning on the date of major surgery, instead of a staging phase.^20^ Patients who died within 30 days of their surgery were defined as operative deaths.

After the staging or surgery phase, patients had a 6‐month initial phase, followed by a continuing phase varying in length between patients depending on survival time. Those patients who died on or before December 31, 2013 had a 6‐month terminal phase ending on the date of death.^21^ Those who survived past 2013 were not considered to have a terminal phase. We allocated costs first to the terminal phase, followed by the staging or surgery phase, the initial phase, and, lastly, the continuing phase, (for those patients who survived long enough to have had all four phases of care). Not all patients contributed to all phases of care, or the full length of phases. For example, a patient who died 10 months after their diagnosis would have 6 months in the terminal phase, 1 month in either the staging or surgery phase, only 3 months in the initial phase, and no continuing phase at all.

Since noncancer control subjects did not have a cancer diagnosis date, they were randomly assigned a “pseudodiagnosis” date matching the diagnosis date of one of the esophageal cancer patients.^19^ Each control patient was assigned to two phases of care—continuing and terminal. Since control patients are not on active cancer treatment, they were not assigned to an initial phase. The terminal phase was defined as the last six months of life, and the continuing phase was defined as the months between the “pseudodiagnosis” date and the start of the terminal phase.

Control subjects in the continuing phase were matched to esophageal cancer patients in the initial phase and in the continuing phase. Health care costs are typically high during the end of life, regardless of cause of death.^22^ To best reflect the costs attributed to cancer during the 6‐month terminal phase, cancer patients who died of their cancer were matched to the continuing control subjects, while cancer patients who died from other causes were matched to the control subjects in the terminal phase.^19^

### 2.5 Cost estimates

We calculated mean total monthly costs for each phase of care for esophageal cancer patients and for the noncancer control subjects.^19^ Total costs were calculated as the sum of Medicare reimbursements (payments made from Medicare to the service provider), coinsurance reimbursements (payments made from a coinsurer to the service provider), and any copayments and deductibles billed to patients. We also determined patient‐liability costs for each cancer patient, which is defined as the total health care costs that are the patient's responsibility, including copayments and deductibles.^20^ Patient‐liability costs may include costs paid by a purchased Medigap policy (insurance sold by private companies to help cover coinsurance, copayment, and deductible costs).^23^

We calculated cancer‐attributable costs within the initial, continuing, and terminal phases for each histology, stage, and treatment subgroups. Cancer‐attributable costs were estimated by subtracting the matched noncancer patient's mean monthly total costs from the esophageal cancer patient's mean monthly total costs.

We converted payments to constant 2018 US dollars by adjusting Part A claims using the CMS Prospective Payment System Hospital Price Index and Part B claims using the Medicare economic index.^24^, ^25^ We present all costs in inflation‐adjusted US dollars.

### 2.6 Statistical analysis

We calculated the monthly total, patient‐liability, and cancer‐attributable costs for each patient and reported the mean monthly estimates and 95% confidence intervals for each phase/AJCC stage/treatment subgroup for both EAC and ESC patients (historic stage is reported in Appendix A: Results). We fit linear regression models using log transformation to estimate the population mean costs. Separate models were fit to each phase, histological subtype, stage at diagnosis, and treatment modality. We only included treatment modality costs for stage/histological groups in which at least 10% of the patients received that treatment. Best supportive care costs are included for all groups. We included calendar year, age, and an interaction term (year × age) as independent terms in the models and used backwards stepwise selection until all terms were statistically significant at the *α* = 0.05 level. A full description of the model parameters and parameter estimates are reported in Appendix B: Parameters. The age variable for the models in the initial and continuing phases was set to the median age at diagnosis (68 years) and the year variable was set to 18 (calendar year 2018).^4^ The median age at death (69 years) was used for cost estimates in the terminal phase.^4^ All analyses were done using SAS 9.4 (Cary, NC).

Institutional Review Board exemption was obtained from Massachusetts General Hospital to review previously collected data.

## 3 RESULTS

### 3.1 Patient cohort characteristics

We identified a total of 8061 esophageal cancer patients diagnosed from 2000 to 2011. An overview of their characteristics is in Table 1. Nearly three‐fourths (74.2%) were male and the median (25th, 75th percentile) age of diagnosis was 75 (70, 80). The majority of patients were White (85.3%). More patients were diagnosed with EAC (62.6%) than with ESC (37.4%). Approximately 23.4% of patients were diagnosed at stage I and 31.9% were diagnosed at stage IV. Among treatment strategies, the treatment with the highest number of patients was chemoradiation, which was received by 37.7% of patients. Only 19.2% of patients received surgery; 21.6% of patients received best supportive care.

**Table 1:** Description of 8061 esophageal cancer patients

Characteristic	N (%)
Male, N (%)	5980 (74.2)
Race/Ethnicity	
White	6876 (85.3)
Black	769 (9.5)
Hispanic	98 (1.2)
Asian	164 (2.0)
Native American/Alaska Native	17 (0.2)
Other	137 (1.6)
2000	572 (7.1)
2001	624 (7.7)
2002	629 (7.8)
2003	686 (8.5)
2004	764 (9.5)
2005	698 (8.7)
2006	716 (8.9)
2007	709 (8.8)
2008	674 (8.4)
2009	685 (8.5)
2010	665 (8.3)
2011	639 (7.9)
Age at diagnosis	
66‐69 y	1780 (22.1)
70‐74 y	2106 (26.1)
75‐79 y	1952 (24.2)
80‐84 y	1318 (16.4)
85+ y	905 (11.2)
Histology and stage at diagnosis	
Adenocarcinoma	
Stage I	1196 (23.7)
Stage II	1046 (20.7)
Stage III	1079 (21.4)
Stage IV	1723 (34.2)
Squamous cell carcinoma	
Stage I	693 (23.0)
Stage II	742 (24.6)
Stage III	731 (24.2)
Stage IV	851 (28.2)
Treatment modality	
Best supportive care	1737 (21.6)
Surgery^a^	1546 (19.2)
Surgery and chemotherapy	634 (7.9)
Chemotherapy	462 (5.7)
Radiation	1281 (15.9)
Chemoradiation	3035 (37.7)
Cause of death^b^	
Esophageal cancer	5418 (74.7)
Operative^c^,	125 (1.7)
All other causes	1711 (23.6)

a 1.0% of all patients received surgery and chemotherapy, 2.0% of all patients received surgery and radiation.

b Percentages represent proportions among the 7254 (90.0%) total deaths during the study period.

c Operative death is defined as death within 30 d of surgery to remove esophageal cancer.

Of the 7254 (90%) of patients who had died by the end of the study period, 5418 (74.7%) died of esophageal cancer, another 125 (1.7%) were operative deaths, and the remaining 1711 (23.6%) died of causes unrelated to cancer. Among all patients who died, the median (25th, 75th percentile) survival time was 8.1 months (3.5, 17.3). Among the patients who died from their cancer, the median (25th, 75th percentile) survival time was 7.5 months (3.4, 14.7). Approximately 69.8% of patients lived 13 months or less, and therefore did not have a continuing phase. Approximately half (50.7%) lived 7 months or less; these patients did not have initial or continuing phases.

The mean (95% CI) phase lengths (in months) for our cohort are reported in Table 2. The mean lengths for the initial, continuing, and terminal phases were 5.47, 26.0, and 4.90 months, respectively, among stage I patients who contributed to the phase. For stage II patients, the mean lengths were 5.40, 22.63, and 5.14 months for the initial, continuing, and terminal phases, respectively. Among stage III patients, the months for the initial, continuing, and terminal phases were 5.20, 18.66, and 4.90 months, respectively. Stage IV patients had mean lengths of 4.75, 13.91, and 4.19 months for the initial, continuing, and terminal phases, respectively.

**Table 2:** Mean monthly cost estimates for each phase by AJCC stage at diagnosis^a^

	Mean phase length^a^ (95% CI)	Total monthly cost (95% CI)	Monthly patient‐liability cost (95% CI)	Monthly cancer‐attributable cost (95% CI)
All stages				
Surgery	0.98 (0.97‐0.99)	$62 760 ($56 541‐$68 980)	$2584 ($2341‐$2827)	b
Staging	0.96 (0.95‐0.97)	$8953 ($8385‐$9485)	$1308 ($1258‐$1358)	b
Initial	5.25 (5.21‐5.29)	$7731 ($7492‐$7970)	$1155 ($1123‐$1187)	$6702 ($6462‐$6943)
Continuing	21.88 (21.33‐22.43)	$2984 ($2814‐$3154)	$390 ($375‐$405)	$1951 ($1780‐$2123)
Terminal	4.70 (4.66‐4.75)	$18 150 ($17 211‐$19 089)	$1433 ($1386‐$1481)	$15 499 ($14 557‐$16 441)
Stage I				
Surgery	0.995 (0.99‐1.00)	$72 914 ($62 160‐$83 667)	$3266 ($2859‐$3673)	b
Staging	0.97 (0.96‐0.98)	$8248 ($7487‐$9029)	$1238 ($1145‐$1330)	b
Initial	5.47 (5.40‐5.54)	$6240 $5861‐$6618)	$948 ($888‐$1006)	$5185 ($4804‐$5565)
Continuing	26.0 (25.0‐27.0)	$2338 ($2160‐$2517)	$314 ($292‐$336)	$1291 ($1108‐$1474)
Terminal	4.90 (4.81‐4.99)	$18 280 ($16 202‐$20 358)	$1334 ($1246‐$1422)	$15 099 ($13 013‐$17 186)
Stage II				
Surgery	0.99 (0.98‐1.00)	$56 124 ($51 503‐$60 745)	$2746 ($2584‐$2907)	b
Staging	0.98 (0.97‐0.99)	$9171 ($7642‐$10 699)	$1275 ($1180‐$1370)	b
Initial	5.4 (5.37‐5.51)	$7478 ($6974‐$7982)	$1106 ($1041‐$1172)	$6444 ($5940‐$6949)
Continuing	22.63 (21.67‐23.60)	$2893 ($2545‐$3240)	$344 ($321‐$367)	$1839 ($1439‐$2189)
Terminal	5.14 (5.06‐5.22)	$20 533 ($17 772‐$23 293)	$1477 ($1336‐$1617)	$17 593 ($14 825‐$20 360)
Stage III				
Surgery	0.97 (0.95‐0.98)	$63 592 ($47 306‐$79 877)	$2632 ($2456‐$2808)	b
Staging	0.95 (0.94‐0.96)	$9249 ($8025‐$10 474)	$1403 ($1266‐$1538)	b
Initial	5.20 (5.11‐5.29)	$8492 ($7978‐$9007)	$1246 ($1181‐$1311)	$7486 ($6970‐$8002)
Continuing	18.66 (17.54‐19.78)	$3394 ($2920‐$3867)	$423 ($392‐$454)	$2389 ($1914‐$2865)
Terminal	4.90 (4.81‐4.98)	$20 599 ($18 268‐$22 929)	$1502 ($1376‐$1628)	$18 143 ($15 809‐$20 476)
Stage IV				
Surgery	0.95 (0.91‐1.00)	$52 908 ($33 950‐$71 865)	$2629 ($2176‐$3083)	b
Staging	0.95 (0.94‐0.96)	$9119 ($8257‐$9981)	$1328 ($1249‐$1407)	b
Initial	4.75 (5.64‐4.86)	$9263 ($8758‐$9 768)	$1404 ($1336‐$1471)	$8252 ($7744‐$8761)
Continuing	13.91 (12.70‐15.11)	$4334 ($3921‐$4747)	$663 ($609‐$719)	$3348 ($2923‐$3772)
Terminal	4.19 (4.12‐4.27)	$15 004 ($14 290‐$15 718)	$1423 ($1381‐$1465)	$12 722 ($11 998‐$13 446)

Abbreviation: AJCC, American Joint Committee on Cancer.

a Among patients who contributed to the phase.

b Staging and Surgery phases have total and patient‐liability costs only.

### 3.2 Mean overall and stage‐specific costs

The mean overall and stage‐specific costs (total, patient‐liability, and cancer‐attributable) by phase are reported in Table 2. Mean (95% CI) monthly total cost estimates for esophageal cancer overall were $8953 ($8385‐$9485) for the staging phase. The mean monthly cancer‐attributable costs were $6702 ($6462‐$6943) for the initial phase, $1951 ($1780‐$3123) for the continuing phase and $15 499 ($14 557‐$16 411) for the terminal phase.

**Figure 1:**
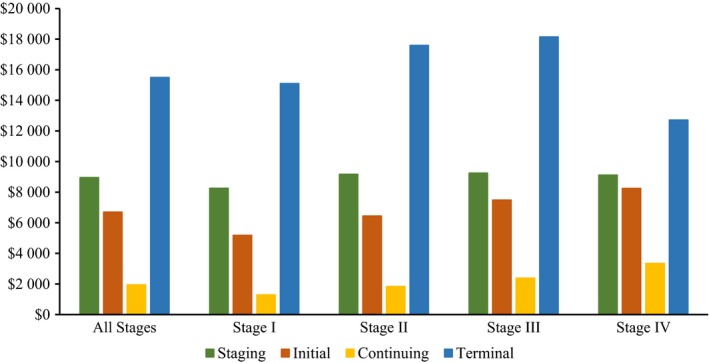
Mean phase‐specific total and cancer‐attributable costs overall and by AJCC stage (cancer‐attributable cost reported for all phases except staging, which are reported as total costs). AJCC, American Joint Committee on Cancer

Stage I patients had mean (95% CI) monthly cancer‐attributable costs of $5185 ($4804‐$5565) in the initial phase, $1291 ($1108‐$1474) in the continuous phase, and $15 099 ($13 013‐$17 186) in the terminal phase. Among stage II patients the mean (95% CI) monthly cancer‐attributable costs were $6444 ($5940‐$6949) in the initial phase, $1839 ($1439‐$2189) in the continuous phase, and $17 593 ($14 825‐$20 360) in the terminal phase. The mean (95% CI) monthly cancer‐attributable costs among stage III patients were $7486 ($6970‐$8002), $2389 ($1914‐42 865), and $18 143 ($15 809‐$20 476) for the initial, continuing, and terminal phases, respectively. For Stage IV patients, the mean (95% CI) monthly cancer‐attributable costs were $8252 ($7744‐$8761), $3348 ($2923‐$3772), and $12 722 ($11 998‐$13 446) for the initial, continuing, and terminal phases, respectively. Cancer‐attributable costs by phase and stage are shown in Figure 1.

### 3.3 Total cost during the staging phase by treatment

The costs during the staging month for patients who received active treatment other than surgery are reported in Appendix Table A2 for AJCC stage and Appendix Table A3 for historic stage. Among EAC patients, the mean (95% CI) total monthly costs during the 1‐month staging phase ranged from $7323 ($4818‐$9828) for stage III patients who received radiation to $12 020 ($7749‐$16 290) for stage II patients who received radiation. These costs remained stable over the study period and were not affected by age in the linear regression models, except in the case of stage II patients who received chemoradiation, whose costs increased over the study period and with age. Patient liability costs (mean (95% CI)) range from $1066 ($807‐$1326) for stage III patients who received radiation to $1375 ($1227‐$1522) for stage I patients who received chemoradiation.

Overall, ESC patients tended to have higher mean total costs than EAC patients. Among ESC patients, the mean (95% CI) total monthly costs ranged from $8362 ($6920‐$9803) for stage I patients who received chemoradiation to $13 885 ($8510‐$19 259) for stage II patients who received radiation. These costs were not affected by age or study period in the linear regressions. Mean (95% CI) patient liability costs ranged from $1321 ($1186‐$1456) for stage I patients who received chemoradiation to $1831 ($1314‐$2349) stage II patients who received radiation.

### 3.4 Total cost during the surgery phase

The mean (95% CI) total surgery phase cost was $62 760 ($56 541‐$68 980). Patients were liable for $2584 ($2341‐$2827) of this cost. Mean (95% CI) total costs ranged from $52 908 ($33 950‐$71 865) for stage IV patients to $72 914 ($62 160‐$83 667) for stage I patients and patient liability costs ranged from $2629 ($2176‐$3083) for stage IV patients to $3266 ($2859‐$3673) for stage I patients (Table 2). Patients who had an operative death had a total cost of $214 244 (95% CI: $180 907‐$247 581) and a patient‐liability cost of $9295 (95% CI: $7420‐$11 171).

Mean (95% CI) total costs for EAC patients in this phase were $70 280 ($58 891‐$81 670), $54 854 ($49 992‐$59 715), $60 599 ($43 103‐$78 096), and $55 328 ($31 995‐$78 660), for stages I, II, III, and IV, respectively (Table 3). Mean (95% CI) total costs for ESC patients in this phase were $84 538 ($54 642‐$114 433), $59 494 ($48 507‐$70 482), $73 274 ($33 327‐$113 220), and $43 992 ($18 566‐$69 418), for stages I, II, III, and IV, respectively.

**Table 3:** Mean monthly cost estimates for surgery by histology and AJCC stage at diagnosis

	Total cost (95% CI)	Patient‐liability cost (95% CI)
Adenocarcinoma		
Stage I	$70 280 ($58 891‐$81 670)	$3010 ($2565‐$3455)
Stage II	$54 854 ($49 992‐$59 715)	$2300 ($2140‐$2459)
Stage III	$60 599 ($43 103‐$78 096)	$2261 ($1995‐$2528)
Stage IV	$55 328 ($31 995‐$78 660)	$2165 ($1357‐$2972)
Squamous cell carcinoma		
Stage I	$84 538 ($54 642‐$114 433)	$3140 ($2230‐$4049)
Stage II	$59 494 ($48 507‐$70 482)	$2536 ($2123‐$2948)
Stage III	$73 274 ($33 327‐$113 220)	$3541 ($867‐$6216)
Stage IV	$43 992 ($18 566‐$69 418)	$1468 ($783‐$2153)

Abbreviation: AJCC, American Joint Committee on Cancer.

### 3.5 Cancer‐attributable costs of treatment during the initial phase

Costs estimates during the 6‐month initial phase for stage and treatment subgroups are reported in Table 4 for AJCC stage and Appendix Table A4 for historic stage. Among EAC patients who received active treatment, the mean (95% CI) cancer‐attributable monthly costs ranged from $2908 ($2188‐$3628) for stage II patients who received surgery, chemotherapy, and radiation to $10 659 ($9743‐$11 575) for stage III patients who received chemoradiation. Mean cancer‐attributable costs in all stages were highest for patients who received chemoradiation. Cancer‐attributable costs were generally unaffected by study period, and either were stable or decreased with increasing age. Mean (95% CI) patient‐liability costs ranged from $454 ($342‐$566) for stage I patients who received surgery to $1693 ($1603‐$1873) for stage III patients who received chemoradiation.

**Table 4:** Mean monthly cost estimates by histology, AJCC stage at diagnosis by treatment modality, and significant predictors of cancer‐attributable costs during the initial phase^a^

	N (%)	Total cost (95% CI)	Patient‐liability cost (95% CI)	Cancer‐attributable cost (95% CI)	Cancer‐attributable cost predictors		
					Year	Age	Year × age
Adenocarcinoma							
Stage I	860						
Best supportive care	245 (28.5)	$2958 ($2426‐$3490)	$392 ($329‐$456)	$1931 ($1366‐$2495)			
Surgery	204 (23.7)	$4054 ($2981‐$5127)	$454 ($342‐$566)	$3086 ($2014‐$4159)			
Radiation	89 (10.4)	$5712 ($4743‐$6680)	$987 ($842‐$1131)	$4506 ($3548‐$6680)			
Chemoradiation	219 (25.5)	$10 146 ($9150‐$11 142)	$1678 ($1480‐$1875)	$9021 ($8020‐$10 022)		−	
Stage II	755						
Best supportive care	79 (10.5)	$2487 ($947‐$4027)	$272 ($110‐$434)	$1453 (−$5 to $2910)	−	−	+
Surgery	109 (14.4)	$4564 ($2000‐$7128)	$552 ($235‐$890)	$3585 ($1027‐$6163)			
Chemoradiation	269 (36.6)	$10 781 ($9845‐$11 718)	$1614 ($1519‐$1710)	$9753 ($8813‐$10 693)		−	
Surgery, chemo, and radiation	160 (21.2)	$3925 ($3220‐$4630)	$561 ($466‐$657)	$2908 ($2188‐$3628)			
Stage III	505						
Best supportive care	55 (8.4)	$1934 ($1046‐$2822)	$175 ($102‐$249)	$947 ($114‐$1780)	+		
Chemoradiation	293 (45.0)	$11 723 ($10 815‐$12 631)	$1693 ($1603‐$1873)	$10 659 ($9743‐$11 575)		−	
Surgery, chemo, and radiation	146 (22.4)	$5502 ($4014‐$6989)	$872 ($703‐$1041)	$4642 ($3177‐$6107)			
Stage IV	591						
Best supportive care	47 (7.6)	$2783 ($1333‐$4233)	$371 ($188‐$554)	$1824 ($388‐$3261)			
Chemotherapy	84 (13.5)	$8076 ($6940‐$9412)	$1269 ($1096‐$1441)	$6929 ($5619‐$8239)			
Chemoradiation	420 (67.5)	$10 349 ($9668‐$11 029)	$1635 ($1544‐$1726)	$9367 ($8677‐$10 057)			
Squamous cell carcinoma							
Stage I	384						
Best supportive care	56 (13.8)	$3275 ($2298‐$4253)	$371 ($188‐$554)	$2167 ($1127‐$3207)			
Radiation	75 (18.5)	$7013 ($5796‐$8229)	$443 ($218‐$901)	$5885 ($4682‐$7089)			
Chemoradiation	206 (50.9)	$10 105 ($9306‐$10 904)	$1553 ($1450‐$1656)	$9061 ($8249‐$9873)		−	
Stage II	440						
Best supportive care	45 (9.5)	$4039 ($1283‐$6795)	$376 ($177‐$576)	$2995 ($206‐$5784)			
Surgery	48 (10.1)	$5703 ($2232‐$9176)	$825 ($315‐$1334)	$4487 ($1003‐$7972)			
Radiation	58 (12.2)	$7601 ($5497‐$9706)	$1277 ($970‐$1583)	$6587 ($4457‐$8718)			
Chemoradiation	261 (54.8)	$10 671 ($9671‐$11 672)	$1633 ($1521‐$1746)	$9599 ($8589‐$10 608)	+		
Stage III	359						
Best supportive care	34 (8.9)	$6026 ($1866‐$10 187)	$706 (−$81 to $1 494)	$5095 ($914‐$9277)			
Radiation	49 (12.8)	$7530 ($5655‐$9405)	$1090 ($894‐$1287)	$6172 ($4234‐$8110)			
Chemoradiation	236 (61.5)	$11 460 ($10 422‐$12 499)	$1775 ($1646‐$1905)	$10 471 ($9422‐$11 519)			
Stage IV	281						
Best supportive care	b	$4097 ($1539‐$6655)	$345 ($125‐$566)	$3290 ($706‐45 874)	+	+	−
Radiation	36 (12.8)	$13 921 ($8725‐$19 117)	$1588 ($887‐$2288)	$12 842 ($7654‐$18 029)			
Chemotherapy	29 (10.3)	$5560 ($4074‐$7046)	$902 ($691‐$1114)	$4097 ($2517‐$5677)			
Chemoradiation	185 (65.8)	$10 125 ($9205‐$11 045)	$1543 ($1433‐$1653)	$9158 ($8236‐$10 078)			

Abbreviation: AJCC, American Joint Committee on Cancer; SEER, Surveillance, Epidemiology, and End Results.

a A positive (+) symbol indicates that the covariate in the regression model has a parameter estimate greater than 0, while a negative (−) symbol indicates that the parameter estimate is less than 0. With the exception of best supportive care costs, treatment modality costs are not shown if less than 10% of patients within a stage/histology group received that treatment.

b N suppressed in accordance with SEER‐Medicare guidelines to mask cells that may be <11 and ensure patient confidentiality.

Overall trends were similar for ESC patients. The mean (95% CI) cancer‐attributable monthly costs ranged $4097 ($2517‐$5677) for stage IV patients who received chemotherapy to $12 842 ($7654‐$18 029) for stage IV patients who received radiation. These costs generally remained stable, except for stage II chemoradiation costs, which increased over the study period. Mean (95% CI) patient‐liability costs ranged from $443 ($218‐$901) for stage I patients who received radiation to $1775 ($1646‐$1905) for stage III patients who received chemoradiation.

### 3.6 Cost by treatment modality during the continuing phase

Monthly treatment cost estimates during the continuing phase are shown in Table 5 for AJCC stage and Appendix Table A5 for historic stage. Overall, monthly costs were lower during the continuing phase than the initial phase. Mean (95% CI) monthly cancer‐attributable costs among EAC patients who received active treatment ranged from $443 ($233‐$653) for stage I patients who received surgery to $4090 ($3420‐$4761) for stage IV patients who received chemoradiation. These costs remained stable in the linear regression models except for those of stage II patients who received surgery or chemoradiation, which decreased with increasing age, and those of stage II patients who received all three treatments, which increased over the study period and with increasing age. Patient‐liability costs (mean (95% CI)) ranged from $201 ($179‐$223) for stage I patients who received surgery to $811 ($721‐$901) for stage IV patients who received chemoradiation.

**Table 5:** Mean monthly cost estimates by histology, AJCC stage at diagnosis by treatment modality, and significant predictors of cancer‐attributable costs during the continuing phase^a^

	N (%)	Total cost (95% CI)	Patient‐liability cost (95% CI)	Cancer‐attributable cost (95% CI)	Cancer‐attributable cost predictors		
					Year	Age	Year × age
Adenocarcinoma							
Stage I	1144						
Best supportive care	322 (28.2)	$2260 ($1868‐$2653)	$266 ($234‐$297)	$1286 ($885‐$1688)			
Surgery	395 (34.5)	$1026 ($583‐$1805)	$201 ($179‐$223)	$443 ($233‐$653)			
Chemoradiation	216 (18.9)	$3647 ($2910‐$4384)	$438 ($375‐$501)	$2553 ($1804‐$3302)			
Stage II	901						
Best supportive care	74 (8.2)	$1851 ($1166‐$2537)	$232 ($122‐$342)	$529 (−$175 to $1234)			
Surgery	158 (17.5)	$1731 ($1221‐$2241)	$237 ($185‐$290)	$769 ($246‐$1292)		−	
Chemoradiation	288 (32.0)	$3622 ($2945‐$4299)	$466 ($391‐$540)	$2616 ($1929‐$3304)		−	
Surgery, chemo, and radiation	236 (26.2)	$2434 ($1172‐$3696)	$276 ($239‐$314)	$1318 ($51‐$2585)	+	+	−
Stage III	623						
Best supportive care	40 (6.4)	$1832 ($1041‐$2623)	$145 ($68‐$222)	$954 ($191‐$1716)			
Chemoradiation	260 (41.7)	$4884 ($3352‐$6417)	$554 ($484‐$623)	$3883 ($2350‐$5416)			
Surgery, chemo, and radiation	172 (27.6)	$2675 ($2219‐$3130)	$387 ($333‐$442)	$1714 ($1246‐$2182)			
Stage IV	387						
Best supportive care	27 (7.0)	$1721 ($436‐$3007)	$252 ($20‐$483)	$563 (−$802 to $1928)			
Chemotherapy	47 (12.1)	$4674 ($3168‐$5191)	$728 ($557‐$898)	$3526 ($2311‐$4743)			
Chemoradiation	251 (64.9)	$6226 ($3326‐$11 654)	$811 ($721‐$901)	$4090 ($3420‐$4761)			
Squamous Cell Carcinoma							
Stage I	443						
Best supportive care	46 (10.4)	$2480 ($1567‐$3393)	$304 ($224‐$368)	$1837 ($555‐$3119)			
Surgery	54 (12.2)	$2970 ($1408‐$4533)	$506 ($93‐$919)	$2011 ($438‐$3585)			
Radiation	58 (13.1)	$2492 ($1853‐$3131)	$356 ($271‐$440)	$1453 ($786‐$2120)			
Chemoradiation	222 (50.1)	$4270 ($1921‐$9492)	$391 ($322‐$460)	$1670 ($1141‐$2198)			
Stage II	556						
Best supportive care	42 (7.6)	$1592 ($839‐$2345)	$153 ($85‐$220)	$438 (−$355 to $1232)	+	+	−
Surgery	71 (12.8)	$3035 ($1492‐$4577)	$355 ($248‐$462)	$2140 ($587‐$3693)			
Chemoradiation	312 (56.1)	$2921 ($2456‐$3387)	$366 ($322‐$411)	$1873 ($1400‐$2346)			
Stage III	354						
Best supportive care	26 (7.3)	$2061 ($814‐$3308)	$209 ($97‐$320)	$578 (−$857 to $2013)			
Chemoradiation	218 (61.6)	$3112 ($2461‐$3763)	$432 ($355‐$510)	$2078 ($1419‐$2737)			
Stage IV	199						
Best supportive care	b	$3052 (−$1277 to $7381)	$186 ($21‐$351)	$2222 (−$2324 to $6768)	−		
Chemotherapy	24 (12.1)	$4534 ($3237‐$5830)	$735 ($481‐$988)	$3624 ($2352‐$4897)			
Chemoradiation	131 (65.8)	$3985 ($3163‐$4827)	$1806 ($816‐$4001)	$2907 ($2017‐$3798)			

Abbreviation: AJCC, American Joint Committee on Cancer; SEER, Surveillance, Epidemiology, and End Results.

a A positive (+) symbol indicates that the covariate in the regression model has a parameter estimate greater than 0, while a negative (−) symbol indicates that the parameter estimate is less than 0. With the exception of best supportive care costs, treatment modality costs are not shown if less than 10% of patients within a stage/histology group received that treatment.

b N suppressed in accordance with SEER‐Medicare guidelines to mask cells that may be <11 and ensure patient confidentiality.

Mean (95% CI) monthly cancer‐attributable costs among ESC patients who received active treatment ranged from $1453 ($786‐$2120) for stage I patients who received radiation to $3624 ($2352‐$4897) for stage IV patients who received chemotherapy. These costs were not affected by year or age.

### 3.7 Terminal phase costs

Monthly treatment cost estimates during the 6‐month terminal phase are shown in Table 6 for AJCC stage and Appendix Table A6 for historic stage. Among EAC patients, mean (95% CI) cancer‐attributable costs ranged from $8505 ($7202‐$9808) for stage I patients who received chemoradiation to $25 662 ($11 751‐$39 573) for stage I patients who received surgery. The cancer‐attributable costs were largely unaffected by year or age in the linear regression models. Mean (95% CI) patient‐liability costs ranged from $1107 ($589‐$1295) for stage II patients who received all three active treatments to $1576 ($1471‐41 680) for stage IV patients who received radiation.

**Table 6:** Mean monthly cost estimates by histology, AJCC stage at diagnosis by treatment modality, and significant predictors of cancer‐attributable costs during the terminal phase^a^

	N (%)	Total cost (95% CI)	Patient‐liability cost (95% CI)	Cancer‐attributable cost (95% CI)	Cancer‐attributable cost predictors		
					Year	Age	Year × age
Adenocarcinoma							
Stage I	887						
Best supportive care	280 (31.6)	$11 530 ($9781‐$13 281)	$901 ($802‐$1001)	$7971 ($6133‐$9809)			
Surgery	110 (12.4)	$31 038 ($17 293‐$44 783)	$1539 ($1102‐$1977)	$25 662 ($11 751‐$39 573)			
Radiation	135 (15.2)	$11 499 ($9866‐$13 133)	$1170 ($998‐$1342)	$9245 ($7557‐$10 933)	−	−	+
Chemoradiation	269 (30.3)	$11 856 ($10 630‐$13 082)	$1389 ($1036‐$1241)	$8505 ($7202‐$9808)			
Stage II	861						
Best supportive care	117 (13.6)	$5843 ($4790‐$6896)	$588 ($447‐$728)	$3323 ($1978‐$4668)			
Surgery	95 (11.0)	$16 777 ($11 109‐$22 446)	$1139 ($887‐$1391)	$13 072 ($7234‐$18 910)			
Radiation	113 (13.1)	$11 117 ($9046‐$13 187)	$1169 ($980‐$1358)	$8530 ($6296‐$10 763)			
Chemoradiation	331 (38.4)	$12 375 ($11 187‐$13 563)	$1311 ($1207‐$1416)	$9437 (48 162‐$10 713)	+	−	
Surgery, chemo, and radiation	133 (15.5)	$20 289 ($13 969‐$26 608)	$1107 ($589‐$1295)	$16 665 ($10 432‐$22 898)			
Stage III	967						
Best supportive care	137 (14.2)	$9505 ($7318‐$11 691)	$736 ($583‐$889)	$7356 ($5171‐$9540)			
Radiation	97 (10.0)	$13 263 ($10 791‐$15 735)	$1425 ($1210‐$1639)	$11 332 ($8778‐$13 885)		−	
Chemoradiation	389 (40.2)	$15 456 ($11 061‐$19 850)	$1304 ($1205‐$1403)	$10 083 ($9030‐$11 136)			
Surgery, chemo, and radiation	156 (16.1)	$17 899 ($13 640‐422 159)	$1295 ($1088‐$1502)	$14 937 (410 563‐$19 312)			
Stage IV	1618						
Best supportive care	367 (22.7)	$11 107 ($10 134‐$12 080)	$988 ($907‐$1068)	$8980 ($7960‐$10 000)			
Radiation	295 (18.2)	$15 742 ($14 453‐$17 032)	$1576 ($1471‐41 680)	$13 533 ($12 254‐$14 813)			
Chemotherapy	215 (13.3)	$11 960 ($10 772‐$13 147)	$1287 ($1177‐$1396)	$9483 ($8089‐$10 876)			
Chemoradiation	672 (41.5)	$11 708 ($11 048‐$12 367)	$1380 ($1319‐$1442)	$9178 ($8425‐$9931)	+	−	
Squamous Cell Carcinoma							
Stage I	604						
Best supportive care	149 (24.7)	$12 755 ($10 140‐$15 348)	$880 ($736‐$1023)	$10 681 ($7996‐$13 368)		−	
Radiation	130 (21.5)	$11 668 ($9764‐$13 574)	$1219 ($1025‐$1413)	$8821 ($6812‐$10 831)		−	
Chemoradiation	253 (41.9)	$12 094 ($10 666‐$13 521)	$1255 ($1104‐$1255)	$9658 (48 152‐$11 163)			
Stage II	639						
Best supportive care	104 (16.3)	$9600 ($6932‐$12 268)	$637 ($487‐$514)	$6885 ($4063‐$9707)			
Radiation	122 (19.1)	$12 381 ($10 599‐$14 163)	$1406 ($1213‐$1599)	$9877 ($7945‐$11 810)			
Chemoradiation	279 (43.7)	$11 801 ($10 617‐$12 985)	$1215 ($1092‐$1338)	$8876 ($7601‐$10 152)		−	+
Stage III	668						
Best supportive care	115 (17.2)	$13 785 ($11 139‐$16 431)	$991 ($832‐$1160)	$11 746 ($9032‐$14 460)			
Radiation	130 (19.5)	$14 407 ($12 333‐$16 482)	$1336 ($1156‐$1517)	$12 165 (49 983‐$14 347)			
Chemoradiation	301 (45.1)	$12 343 ($11 160‐$13 526)	$1306 ($1199‐$1414)	$9908 ($8645‐$11 171)	+	−	
Stage IV	786						
Best supportive care	180 (22.9)	$13 038 ($11 395‐$14 682)	$1074 ($948‐$1201)	$10 956 ($9300‐$12 613)	−		
Radiation	199 (25.3)	$17 789 ($15 553‐$20 024)	$1590 ($1410‐$1768)	$15 589 ($13 304‐$17 874)			
Chemoradiation	301(38.3)	$13 581 ($12 431‐$14 732)	$1395 ($1291‐$1498)	$11 443 ($10 245‐$12 641)			

Abbreviation: AJCC, American Joint Committee on Cancer.

a A positive (+) symbol indicates that the covariate in the regression model has a parameter estimate greater than 0, while a negative (−) symbol indicates that the parameter estimate is less than 0. Treatment modality costs are not shown if less than 10% of patients within a stage/histology group received that treatment.

Among ESC patients, mean (95% CI) cancer‐attributable costs ranged from $8821 ($6812‐$10 831) for stage I patients who received radiation to $15 589 ($13 304‐$17 874) for stage IV patients who received radiation. Costs were largely unaffected by year and either were stable or decreased with age in the linear regressions. Mean (95% CI) patient‐liability costs ranged $1215 ($1092‐$1338) for stage II patients who received chemoradiation to $1590 ($1410‐$1768) for stage IV patients who received radiation.

### 3.8 Cancer‐attributable cost of best supportive care

Although patients who received best supportive care did not incur costs of actively treating cancer, we allocated their costs into the same phases to enable comparison across modalities. For EAC best supportive care patients, mean (95% CI) total costs during the staging phase ranged from $3974 ($2064‐$5884) to $6920 ($3700‐$10 171). The mean (95% CI) monthly cancer‐attributable costs during the initial phase ranged from $947 ($114‐$1780) to $1931 ($1366‐$2495) and those during the continuing phase ranged from $529 (−$175 to $1234) to $1286 ($885‐$1688). Mean (95% CI) monthly cancer‐attributable costs during the terminal phase for patients receiving best supportive care ranged from $3323 ($1978‐$4668) to $8980 ($7960‐$10 000). Cancer‐attributable costs were largely unaffected by year and age in the linear regression models, with the exception of stages III patients during the initial phase, where costs increased with year.

Costs for ESC patients who received best supportive care overall were higher than EAC patients. The mean (95% CI) total costs for ESC best supportive care patients during the staging phase ranged from $3231 ($896‐$5566) to $11 097 ($6761‐$15 432). The mean (95% CI) monthly cancer‐attributable costs during the initial phase ranged from $2167 ($1127‐$3207) to $5095 ($914‐$9277) and those during the continuing phase ranged from $438 (−$355 to $1232) to $2222 (−$2324 to $6768). Mean (95% CI) monthly cancer‐attributable costs during the terminal phase for patients receiving best supportive care ranged from $6885 ($4063‐$9707) to $11 746 ($9032‐$14 460). Cancer‐attributable costs were largely unaffected by year and age in the linear regression models, with the exception of stages II and IV patients, where costs increased with year and increasing age during the initial phases and stage IV patients, where costs decreased with year during the continuing and terminal phases.

## 4 DISCUSSION

Our study estimated phase‐specific costs for esophageal cancer patients in the SEER‐Medicare database by age, year, stage, histology, and treatment groups. Our results show that these costs vary widely within each stage and histology subgroup.

Our cost analysis demonstrated the expected pattern of high costs during the 1‐month staging and surgery phases, decreasing over the initial and continuing phases and increasing during the 6‐month terminal phase, for patients diagnosed at stages I and II. For stage III and IV patients, mean costs increased from the staging to initial phases. This is possibly due to the high rates of chemoradiation among these patients, which would incur multiple visits over the course of the phase. Costs were highest in the terminal phase; these high costs are consistent with studies that show high rates of aggressive care, such as hospitalizations and intensive care admissions, among cancer patients during the last months of life.^26^, ^27^, ^28^, ^29^ Our linear regression models found that cancer‐attributable costs remained stable over time for the majority of active treatment subgroups. Such costs were either unaffected by patient age or observed as decreasing with increasing age, except in the case of stage II EAC patients who received all treatments, which showed increasing costs with age in the continuing phase.

Mean cancer‐attributable costs among stage I and stage II EAC patients during the 6‐month initial phase and during the continuing phase were lowest for those who received surgery and highest for those who received chemoradiation. This is to be expected as chemoradiation patients receive their treatment over several months, while surgery patients receive treatment within 1 month. Among ESC patients, those diagnosed with stages I and II and who received chemoradiation had the highest mean cancer‐attributable costs in the initial phase. In the initial and continuing phases, chemoradiation was the most common treatment modality for stage III and IV EAC and ESC patients and also had the highest cancer‐attributable costs. Mean terminal phase cancer‐attributable costs were high in all histology, stage, and treatment subgroups, with the highest among stage IV ESC patients.

However, among stage I and II ESC patients, mean cancer‐attributable costs in the continuing phase were higher for patients who received surgery, at $2011 and $2140, respectively, than patients who received chemoradiation, at $1670 and $1873, respectively. Also, these patients continuing phase cancer‐attributable costs were much higher than those of stage I and II EAC patients, which were $443 and $769, respectively. In addition, mean total costs in the surgery phase for ESC patients were generally higher than EAC patients, ranging from $59 494‐$84 538 for ESC stages I‐III vs $54 854‐$70 280 for EAC stages I‐III. These differences are possibly due to the higher rates of postoperative complication rates found in ESC patients, including pulmonary complications requiring longer hospital stays.^30^, ^31^, ^32^ These complications could potentially last over several months.

Esophageal cancer is often diagnosed at an advanced stage, where curative treatment is no longer an option.^5^ As a result, targeted screening programs have become an increasingly important topic of discussion among researchers and policy makers.^33^ A potential shift in stage distribution would affect treatment patterns, since those diagnosed earlier have more treatment options available. In addition, newer treatment modalities are being developed, such as MIE, which will provide patients with alternatives to open esophagectomy.^9^, ^10^ As a result, the rates at which patients choose specific treatments will likely shift, causing changes in resource utilization, such as a potential reduction in hospitalizations for the postoperative complications associated with invasive surgery.^34^ A detailed understanding of recent costs associated with esophageal cancer treatment, based on studies such as ours, is necessary for researchers who hope to predict future cancer care costs.

While phase‐specific costs have been published for several cancer sites, we found a lack of cost estimates with this level of detail for esophageal cancer in the US^21^, ^35^ One phase‐specific cost study by Kaye (2018) included esophageal cancer as one of 10 different cancers.^15^ The Kaye study estimated a mean annual total cost of $20 433 (mean $1703/month) for the initial phase and $18 760 (mean $1563/month) for the terminal phase. Our cost estimates for the initial and terminal phases appear higher, with mean monthly total costs ranging from $6240‐$9263 for the initial phase and $15 004‐$18 280 for the terminal phase. This difference can likely be attributed to the phase definitions employed by the Kaye study, which set the initial and terminal phase lengths to 12 months for all cancer sites. We defined the lengths of both phases as 6 months and believe these shorter lengths allow us to more accurately estimate costs of these two distinct phases, including the high costs seen during a patient's last months of life. In addition, our study allocates costs of the month starting at diagnosis to a separate staging phase, for non‐cancer patients, and costs of the month starting on the date of surgery to a surgery phase. In the Kaye study, these costs are part of the initial phase.

Our study's strength is that it provides detailed cost estimates for esophageal cancer at the histology, stage, and treatment level. There were, however, several limitations. We used SEER‐Medicare data, which are limited to patients aged 65 or older who were diagnosed in specific geographic areas; this patient base may not be generalizable to the entire US population. Although one could extrapolate results for younger esophageal cancer patients from our linear regression models, such an approach has not been validated for a younger population. In addition, the majority, or 60%, of esophageal cancer patients are diagnosed at ages over 65.^4^ Moreover, we were unable to determine how much of the patient liability costs were paid by Medigap coverage versus out‐of‐pocket by the patient. Furthermore, since we do not have claims information for patients who received their care through an HMO, our cost estimates may not be applicable to such patients. Lastly, it is possible that some costs were misclassified based on our phase of care definitions. For example, patients who died in early 2014 may have had terminal costs classified as initial or continuing costs because we did not have claims beyond December 31, 2013.

In conclusion, our detailed cost estimate analysis demonstrates that the economic burden of esophageal cancer is significant across all stage and histology subgroups. As treatment regimens continue to rapidly evolve, these up‐to‐date cost estimates can serve as an important baseline for healthcare systems and cancer control policy leaders to guide resource allocation in the present and future.

## CONFLICT OF INTEREST

The authors declare that there is no conflict of interest.

## Data Availability

The data that support the findings of this study are available from the SEER‐Medicare linked database administered by the National Cancer Institute. The data are not publicly available due to privacy or ethical restrictions.
