# Supplementary material for: Esophageal cancer treatment costs by phase of care and treatment modality, 2000‐2013
Source: Cancer Med. 2019 Jul 26;8(11):5158–72. doi: 10.1002/cam4.2451 (PMC6718574; doi:10.1002/cam4.2451)
Supplement: Supplementary file 1 [file CAM4-8-5158-s001.docx]

**Table 1. SEER-Medicare Claims Codes**

| Variable | Source | Codes |
| --- | --- | --- |
| Adenocarcinoma | SEER ICD-0-3 codes | 8050, 8140-8147, 8160-8162, 8180-8221, 8250-8507, 8514, 8520-8551, 8560, 8570-8574, 8576, 8940-8941 |
| Squamous cell carcinoma | SEER ICD-0-3 codes | 8070-8078, 8083, 8084 |
| Surgery (including local endoscopic therapy) | inpatient, outpatient, physician, hospice, home health, or durable medical equipment claims | 43100, 43101, 43107, 43108, 43112, 43113, 43116, 43117, 43118, 43119, 43121, 43122, 43123, 43124, 43217, 43216, 43228, 43250, 43251, 43257, 43258, 96570, 96571, 96570, 96571, 42.33, 42.40-42.42, 42.50-42.59, 42.60-42.69, 43.5, 43.99 |
| Radiation | inpatient, outpatient, physician, hospice, home health, or durable medical equipment claims | ICD-9-CM: V58.0, 92.21-92.29  HCPCS: code S8049  CPT: 77000-77999 or 79000-79999  Revenue Center: 0330, 0333, 0339 |
| Chemotherapy | inpatient, outpatient, physician, hospice, home health, or durable medical equipment claims | ICD-9-CM: V58.1, V58.11, 99.25  HCPCS: C1166, C1167, C1178, C9110, C9205, C9207, C9213-C9216, C9411, C9414-C9419, C9420-C9438, G0355, G0356, G0359-G0362, J7150, J8500-J8799, J8999-J9999, Q0083-Q0085, S9325-S9329, S9330-S9379, or S9494-S9497  CPT: 96400-96546  Revenue Center 0331, 0332, 0335 |

**Table 2.** Significant predictors of costs during the staging phase and monthly costs by histology, stage at diagnosis, and treatment modality†

|  |  |  |  | *Total Cost Predictors* | | |
| --- | --- | --- | --- | --- | --- | --- |
|  | N (%) | Total cost (95% CI) | Patient-liability cost (95% CI) | Year | Age | Year*age |
| *Adenocarcinoma* |  |  |  |  |  |  |
| **Stage I** | 599 |  |  |  |  |  |
| Best supportive care | 250 (41.7) | $5,611 ($4,524-$6,697) | $796 ($676-$916) |  |  |  |
| Radiation | 97 (16.2) | $9,675 ($6,857-$12,493) | $1,348 ($987-$1,709) |  |  |  |
| Chemoradiation | 226 (37.7) | $8,214 ($6,910-$9,518) | $1,375 ($1,227-$1,522) |  |  |  |
| **Stage II** | 473 |  |  |  |  |  |
| Best supportive care | 82 (17.3) | $3,974 ($2,064-$5,884) | $519 ($357-$680) |  | **+** |  |
| Radiation | 76 (16.1) | $12,020 ($7,749-$16,290) | $1,290 ($1000-$1,581) |  |  |  |
| Chemoradiation | 288 (60.9) | $8,210 ($6,810-$9,611) | $1,339 ($1,192-$1,486) | **+** | **+** | **-** |
| **Stage III** | 446 |  |  |  |  |  |
| Best supportive care | 63 (14.1) | $6,920 ($3,700-$10,171) | $1,125 ($409-$1,840) |  | **+** |  |
| Radiation | 51 (11.4) | $7,323 ($4,818-$9,828) | $1,066 ($807-$1,326) |  |  |  |
| Chemoradiation | 303 (67.9) | $7,969 ($6,921-$9,019) | $1,357 ($1,235-$1,479) |  |  |  |
| **Stage IV** | 675 |  |  |  |  |  |
| Best supportive care | 63 (9.3) | $6,817 ($3,583-$10.052) | $719 ($505-$934) |  |  |  |
| Chemotherapy | 105 (15.6) | $10,779 ($5,861-$15,698) | $1,360 ($1,035-$1,686) |  |  |  |
| Chemoradiation | 462 (68.4) | $8,453 ($7,351-$9,556) | $1,352 ($1,240-$1,463) |  |  |  |
| *Squamous Cell Carcinoma* |  |  |  |  |  |  |
| **Stage I** | 379 |  |  |  |  |  |
| Best supportive care | 65 (17.2) | $11,097 ($6,761-$15,432) | $1,535 ($831-$2,238) |  | **+** |  |
| Radiation | 81 (21.4) | $10,119 ($6,363-$13,875) | $1,578 ($1,017-$2,138) |  | **+** |  |
| Chemoradiation | 220 (58.1) | $8,362 ($6,920-$9,803) | $1,321 ($1,186-$1,456) |  |  |  |
| **Stage II** | 413 |  |  |  |  |  |
| Best supportive care | 49 (11.9) | $6,317 (-$428-$13,061) | $567 ($214-$920) |  |  |  |
| Radiation | 72 (17.4) | $13,885 ($8,510-$19,259) | $1,831 ($1,314-$2,349) |  |  |  |
| Chemoradiation | 278 (67.3) | $9,073 ($7,235-$10,913) | $1,498 ($1,309-$1,688) |  |  |  |
| **Stage III** | 356 |  |  |  |  |  |
| Best supportive care | ‡ | $3,231 ($896-$5,566) | $468 ($233-$704) |  |  |  |
| Radiation | 57 (16.0) | $12,113 ($8,346-$15,880) | $1,739 ($1,247-$2,231) |  |  |  |
| Chemoradiation | 252 (70.8) | $11,343 ($7,871-$14,815) | $1,673 ($1,344-$2,001) |  |  |  |
| **Stage IV** | 317 |  |  |  |  |  |
| Best supportive care | 28 (8.8) | $8,561 ($2,049-$15,073) | $776 ($414-$1,137) |  |  |  |
| Radiation | 47 (14.8) | $11,959 ($7,299-$16,620) | $1,403 ($1,115-$1,690) |  |  |  |
| Chemotherapy | 35 (11.0) | $10,568 ($6,827-$14,310) | $1,585 ($1,219-$1,950) |  |  |  |
| Chemoradiation | 207 (65.3) | $9,193 ($7,760-$10,627) | $1,455 ($1,259-$1,651) |  |  |  |

†The directions of significant predictors are shown for linear regression models of total costs. A positive (+) symbol indicates that the covariate in the regression model has a parameter estimate greater than 0, while a negative (-) symbol indicates that the parameter estimate is less than 0. With the exception of best supportive care costs, treatment modality costs are not shown if less than 10% of patients within a stage/histology group received that treatment.

‡N suppressed in accordance with SEER-Medicare guidelines to mask cells that may be <11 and ensure patient confidentiality.

**Table 3.** Significant predictors of costs during the staging phase and monthly costs by histology, stage at diagnosis, and treatment modality†

|  |  |  |  | *Total Cost Predictors* | | |
| --- | --- | --- | --- | --- | --- | --- |
|  | N (%) | Total cost (95% CI) | Patient-liability cost (95% CI) | Year | Age | Year*age |
| *Adenocarcinoma* |  |  |  |  |  |  |
| **Local** | 712 |  |  |  |  |  |
| Best supportive care | 275 (38.6) | $5,451 ($4,432-$6,469) | $778 ($667-$889) |  | **+** |  |
| Radiation | 120 (16.9) | $10,237 ($7,512-$12,962) | $1,346 ($1,038-$1,654) |  |  |  |
| Chemoradiation | 289 (40.6) | $8,045 (6,913-$9,177) | $1,343 ($1,217-$1,470) | **+** | **+** | **-** |
| **Regional** | 711 |  |  |  |  |  |
| Best supportive care | 105 (14.8) | $5,548 ($3,308-$7,788) | $828 (4393-$1,263) |  | **+** |  |
| Radiation | 91 (12.8) | $10,195 ($6,984-$13,405) | $1,201 ($966-$1,436) |  |  |  |
| Chemoradiation | 472 (66.4) | $5,300 ($4,564-$6,153) | $1,345 ($1,236-$1,454) |  | **-** |  |
| **Distant** | 762 |  |  |  |  |  |
| Best supportive care | 76 (10.0) | $6,593 ($3,783-$9,403) | $726 ($534-$918) | **-** | **-** | **+** |
| Chemotherapy | 114 (15.0) | $10,670 ($6,104-$15,237) | $1,337 ($1,034-$1,639) |  |  |  |
| Chemoradiation | 516 (67.7) | $8,443 ($7,434-$9,453) | $1,369 ($1,264-$1,474) |  |  |  |
| *Squamous Cell Carcinoma* |  |  |  |  |  |  |
| **Local** | 479 |  |  |  |  |  |
| Best supportive care | 73 (15.2) | $12,628 ($6,996-$18,260) | $1,517 ($878-$2,156) |  | **+** |  |
| Radiation | 101 (21.1) | $10,928 ($7,598-$14,257) | $654 ($450-$950) |  | **+** |  |
| Chemoradiation | 291 (60.8) | $8,400 ($7,102-$9,700) | $1,384 ($1,213-$1,556) |  |  |  |
| **Regional** | 560 |  |  |  |  |  |
| Best supportive care | 66 (11.8) | $3,354 ($1,702-$5,007) | $497 ($274-$721) |  |  |  |
| Radiation | 89 (15.9) | $14,225 ($9,745-$18,706) | $1,921 ($1,452-$2,390) |  |  |  |
| Chemoradiation | 390 (69.6) | $8,845 ($7,787-$9,903) | $1,448 ($1,337-$1,558) |  |  |  |
| **Distant** | 418 |  |  |  |  |  |
| Best supportive care | 40 (9.6) | $5,969 ($1,429-$10,508) | $560 ($294-$826) |  |  |  |
| Radiation | 65 (15.6) | $10,417 ($6,831-$14,004) | $1,378 ($1,088-$1,668) |  |  |  |
| Chemotherapy | 39 (9.3) | $11,267 ($6,825-$15,710) | $1,580 ($1,211-$1,949) |  |  |  |
| Chemoradiation | 274 (65.6) | $11,721 ($8,268-$15,173) | $1,677 ($1,355-$2,000) |  |  |  |

†The directions of significant predictors are shown for linear regression models of total costs. A positive (+) symbol indicates that the covariate in the regression model has a parameter estimate greater than 0, while a negative (-) symbol indicates that the parameter estimate is less than 0. With the exception of best supportive care costs, treatment modality costs are not shown if less than 10% of patients within a stage/histology group received that treatment.

**Table 4.** Significant predictors of cancer-attributable costs during the initial phase and monthly costs by histology, stage at diagnosis, and treatment modality†

|  |  |  |  |  | *Cancer-attributable Cost Predictors* | | |
| --- | --- | --- | --- | --- | --- | --- | --- |
|  | N (%) | Total Cost  (95% CI) | Patient-liability cost  (95% CI) | Cancer-attributable cost  (95% CI) | Year | Age | Year*age |
| *Adenocarcinoma* |  |  |  |  |  |  |  |
| **Local** | 1,059 |  |  |  |  |  |  |
| Best supportive care | 269 (25.4) | $2,899 ($2,400-$3,397) | $379 ($320-$438) | $1,873 ($1,346-$2,400) |  |  |  |
| Surgery | 257 (24.3) | $4,208 ($2,899-$5,517) | $503 ($347-$659) | $3,252 ($1,945-$4,559) |  |  |  |
| Radiation | 109 (10.3) | $6,267 ($4,912-$7,621) | $1,065 ($894-$1,236) | $5,083 ($3,718-$6,448) |  |  |  |
| Chemoradiation | 278 (26.3) | $10,030 ($9,127-$10,934) | $1,630 ($1,471-$1,790) | $8,931 ($8,022-$9,840) |  | **-** |  |
| **Regional** | 1,106 |  |  |  |  |  |  |
| Best supportive care | 97 (8.8) | $2,209 ($929-$3,489) | $223 ($92-$354) | $1,157 (-$46-$2,360) |  |  |  |
| Chemoradiation | 452 (40.9) | $11,497 ($10,772-$12,223) | $1,681 ($1,608-$1,755) | $10,432 ($9,702-$11,162) |  | **-** |  |
| Surgery, chemo, and radiation | 253 (22.9) | $4,889 ($3,964-$5,814) | $758 ($647-$869) | $3,955 ($3,037-$4,874) |  |  |  |
| **Distant** |  |  |  |  |  |  |  |
| Best supportive care | 62 (8.4) | $2,728 ($1,537-$3,918) | $348 ($198-$498) | $1,814 ($637-$2,992) |  |  |  |
| Chemotherapy | 95 (12.9) | $8,412 ($7,111-$9,712) | $1,274 ($1,114-$1,434) | $7,306 ($6,025-$8,587) |  |  |  |
| Chemoradiation | 473 (64.0) | $10,444 ($9,800-$11,088) | $1,637 ($1,552-$1,723) | $9,469 ($8,816-$10,122) |  |  |  |
| *Squamous Cell Carcinoma* |  |  |  |  |  |  |  |
| **Local** | 549 |  |  |  |  |  |  |
| Best supportive care | 65 (11.8) | $3,272 ($2,365-$4,179) | $376 ($261-$2,530) | $2,200 ($1,238-$3,162) |  |  |  |
| Radiation | 94 (17.1) | $6,700 ($5,664-$7,736) | $1,147 ($1,001-$1,292) | $6,700 ($5,664-$6,603) | + | + | **-** |
| Chemoradiation | 277 (50.5) | 10,078 ($9,362-$10,793) | $1,542 ($1,444-$1,641) | $9,036 ($8,315-$9,757) |  | - |  |
| **Regional** | 652 |  |  |  |  |  |  |
| Best supportive care | 63 (9.7) | $5,805 ($2,823-$8,788) | $631 ($180-$1,081) | $4,804 ($1,797-$7,811) |  |  |  |
| Radiation | 76 (11.7) | $7,940 ($6,026-$9,854) | $1,200 ($946-$1,455) | $6,780 ($4,826-$8,735) |  |  |  |
| Chemoradiation | 370 (56.8) | $11,442 ($10,540-$12,343) | $1,744 ($1,643-$1,845) | $10,414 ($9,504-$11,326) | + |  |  |
| **Distant** | 391 |  |  |  |  |  |  |
| Best supportive care | 35 (9.0) | $3,028 ($1,265-$4,791) | $254 ($101-$406) | $2,125 ($327-$3,923) |  |  |  |
| Radiation | 54 (13.8) | $12,174 ($8,464-$15,883) | $1,498 ($1,010-$1,987) | $11,025 ($7,309-$14,741) |  |  |  |
| Chemoradiation | 246 (62.9) | $10,047 ($9,293-$10,799) | $1,568 ($1,474-$1,662) | $9,053 ($8,296-$9,810) |  |  |  |

†A positive (+) symbol indicates that the covariate in the regression model has a parameter estimate greater than 0, while a negative (-) symbol indicates that the parameter estimate is less than 0. With the exception of best supportive care costs, treatment modality costs are not shown if less than 10% of patients within a stage/histology group received that treatment.

**Table 5.** Significant predictors of cancer-attributable costs during the continuing phase and monthly costs by histology, stage at diagnosis, and treatment modality†

|  |  |  |  |  | *Cancer-attributable Cost Predictors* | | |
| --- | --- | --- | --- | --- | --- | --- | --- |
|  | N (%) | Total Cost  (95% CI) | Patient-liability cost  (95% CI) | Cancer-attributable cost  (95% CI) | Year | Age | Year*age |
| *Adenocarcinoma* |  |  |  |  |  |  |  |
| **Local** | 1,413 |  |  |  |  |  |  |
| Best supportive care | 351 (24.8) | $2,195 ($1,830-$2,560) | 259 ($229-$289) | $1,186 ($813-$1,559) |  |  |  |
| Surgery | 477 (33.8) | $1,493 ($1,265-$1,640) | 236 ($195-$276) | $469 ($273-$666) | + | + | - |
| Chemoradiation | 288 (20.4) | $3,540 ($2,911-$4,169) | $509 ($369-$509) | $2,498 ($1861-$3,135) |  |  |  |
| **Regional** | 1,152 |  |  |  |  |  |  |
| Best supportive care | 71 (6.2) | $2,056 ($1,310-$2,802) | $206 ($98-$314) | $930 ($164-$1,696) |  |  |  |
| Surgery | 128 (11.1) | $1,800 ($1,300-$2,301) | $244 ($205-$283) | $702 ($184-$1,219) |  |  |  |
| Chemoradiation | 433 (37.6) | $4,360 ($3,370-$5,351) | $510 ($459-$561) | $3,335 ($2,341-$4,329) |  |  |  |
| Surgery, chemo, and radiation | 323 (28.0) | $2,213 ($1,927-$2,499) | $323 ($289-$357) | $1,196 ($893-$1,498) |  |  |  |
| **Distant** | 474 |  |  |  |  |  |  |
| Best supportive care | 39 (8.2) | $1,658 ($710-$2,606) | $237 ($70-$404) | $593 ($-383-$1,568) |  |  |  |
| Chemotherapy | 52 (11.0) | $4,699 ($3,562-$5,836) | $725 ($565-$885) | $3,644 ($2,472-$4,816) | - | - | + |
| Chemoradiation | 292 (61.6) | $4,912 ($4,306-$5,519) | $781 ($697-$864) | $4,011 ($3,398-$4,623) |  |  |  |
| *Squamous Cell Carcinoma* |  |  |  |  |  |  |  |
| **Local** | 608 |  |  |  |  |  |  |
| Best supportive care | 49 (8.1) | $2,393 ($1,530-$3,256) | $302 ($226-$378) | $1,022 ($90-$3,256) |  |  |  |
| Surgery | 80 (13.2) | $2,900 ($1,762-$4,038) | $442 ($165-$718) | $2,009 ($865-$3,152) |  |  |  |
| Radiation | 74 (12.2) | $2,906 ($2,034-$3,778) | $385 ($298-$472) | $1,865 ($972-$2,759) | - | - |  |
| Chemoradiation | 314 (51.6) | $2,646 ($2,241-$3,051) | $376 ($323-$430) | $2,005 ($1,169-$2,005) |  |  |  |
| **Regional** | 651 |  |  |  |  |  |  |
| Best supportive care | 53 (8.1) | $2,000 ($1,217-$2,783) | $175 ($109-$242) | $739 ($-112-$1,590) |  |  |  |
| Chemoradiation | 386 (59.3) | $3,039 ($2,572-$3,507) | $392 ($343-$440) | $1,959 ($1,485-$2,433) |  |  |  |
| **Distant** | 277 |  |  |  |  |  |  |
| Best supportive care | 20 (7.2) | $1,788 ($-20-$3,598) | $159 ($52-$266) | $816 (-$1,139-$2,771) | + | - |  |
| Chemoradiation | 181 (65.3) | $3,948 ($3,234-$4,662) | $572 ($481-$663) | $2,931 ($2,186-$3,676) |  |  |  |

†A positive (+) symbol indicates that the covariate in the regression model has a parameter estimate greater than 0, while a negative (-) symbol indicates that the parameter estimate is less than 0. With the exception of best supportive care costs, treatment modality costs are not shown if less than 10% of patients within a stage/histology group received that treatment.

**Table 6.** Significant predictors of cancer-attributable costs during the terminal phase and monthly costs by histology, stage at diagnosis, and treatment modality†

|  |  |  |  |  | *Cancer-attributable Cost Predictors* | | |
| --- | --- | --- | --- | --- | --- | --- | --- |
|  | N (%) | Total Cost  (95% CI) | Patient-liability cost  (95% CI) | Cancer-attributable cost  (95% CI) | Year | Age | Year*age |
| *Adenocarcinoma* |  |  |  |  |  |  |  |
| **Local** | 1,092 |  |  |  |  |  |  |
| Best supportive care | 314 (28.8) | $10,889 ($9,301-$12,477) | $857 ($764-$951) | $7,463 ($5,801-$9,125) |  |  |  |
| Surgery | 144 (13.2) | $27,437 ($16,679-$38,196) | $1,409 ($1,062-$959) | $22,736 ($11,867-$33,606) |  |  |  |
| Radiation | 166 (15.2) | $11,417 ($9,868-$12,965) | $1,152 ($1,002-$1,303) | $9,130 ($7,532-$10,728) |  |  |  |
| Chemoradiation | 341 (31.2) | $11,684 ($10,619-$12,748) | $1,168 ($1,073-$1,263) | $8,456 ($7,329-$9,582) |  |  |  |
| **Regional** | 1,443 |  |  |  |  |  |  |
| Best supportive care | 187 (13.0) | $7,930 ($6,300-$9,561) | $661 ($537-$784) | $5,497 ($3,776-$7,218) |  |  |  |
| Radiation | 156 (10.8) | $12,598 ($10,695-$14,501) | $1,338 ($1,167-$1,510) | $10,303 ($8,281-$12,324) |  | **-** |  |
| Chemoradiation | 568 (39.4) | $12,616 ($11,754-$13,478) | $1,292 ($1,212-$1,372) | $9,864 ($8,919-$10,808) | **+** |  |  |
| Surgery, chemo, and radiation | 249 (17.3) | $18,614 ($14,696-$22,531) | $1,200 ($1,054-$1,347) | $15,399 ($11,461-$19,337) |  |  |  |
| **Distant** | 1,782 |  |  |  |  |  |  |
| Best supportive care | 398 (22.3) | $10,986 ($10,062-$11,909) | $981 ($903-$1,059) | $8,898 ($7,939-$11,909) |  |  |  |
| Radiation | 316 (17.7) | $15,361 ($14,133-$16,589) | $1,552 ($1,450-$1,654) | $13,166 ($11,944-$14,388) |  |  |  |
| Chemotherapy | 229 (12.9) | $12,001 ($10,850-$13,152) | $1,289 ($1,183-$1,395) | $9,605 ($8,262-$10,948) |  |  |  |
| Chemoradiation | 396 (43.3) | $11,816 ($11,187-$12,445) | $1,387 ($1,330-$1,447) | $9,328 ($8,616-$10,041) | **+** | **-** |  |
| *Squamous Cell Carcinoma* |  |  |  |  |  |  |  |
| **Local** | 749 |  |  |  |  |  |  |
| Best supportive care | 173 (23.1) | $12,149 ($9,793-$14,504) | $824 ($696-$952) | $10,099 ($47,671-$12,526) |  |  |  |
| Radiation | 155 (20.7) | $11,829 ($10,017-$13,640) | $1,279 ($1,091-$1,587) | $8,783 ($6,837-$10,730) |  |  |  |
| Chemoradiation | 317 (42.3) | $11,981 ($10,730-$13,232) | $1,231 ($1,100-$1,362) | $9,365 ($8,034-$7,432) |  |  |  |
| **Regional** | 977 |  |  |  |  |  |  |
| Best supportive care | 160 (16.4) | $11,960 ($9,742-$14,176) | $854 ($715-$994) | $9,480 ($7,148-$11,813) |  |  |  |
| Radiation | 191 (19.6) | $13,699 ($12,203-$15,195) | $1,383 ($1,242-$1,524) | $11,456 ($9,870-$13,043) |  | **-** |  |
| Chemoradiation | 431 (44.1) | $12,259 ($11,273-$13,246) | $1,270 ($1,175-$1,365) | $9,584 ($8,528-$10,639) | **+** | **-** |  |
| **Distant** | 955 |  |  |  |  |  |  |
| Best supportive care | 213 (22.3) | $13,100 ($11,515-$14,685) | $1,051 ($934-$1,168) | $11,021 ($9,421-$12,620) |  |  |  |
| Radiation | 233 (24.4) | $17,009 ($15,002-$19,016) | $1,523 ($1,361-$1,686) | $14,863 ($12,818-$16,909) |  |  |  |
| Chemoradiation | 384 (40.2) | $13,147 ($12,152-$14,143) | $1,379 ($1,288-$1,470) | $11,008 ($9,968-$12,048) |  |  |  |

†A positive (+) symbol indicates that the covariate in the regression model has a parameter estimate greater than 0, while a negative (-) symbol indicates that the parameter estimate is less than 0. With the exception of best supportive care costs, treatment modality costs are not shown if less than 10% of patients within a stage/histology group received that treatment.
