# Supplementary material for: Esophageal cancer treatment costs by phase of care and treatment modality, 2000‐2013
Source: Cancer Med. 2019 Jul 26;8(11):5158–72. doi: 10.1002/cam4.2451 (PMC6718574; doi:10.1002/cam4.2451)
Supplement: Supplementary file 2 [file CAM4-8-5158-s002.docx]

**Description of model parameters**

We fit linear regression models using log transformation within the stage/histology subgroups for each treatment strategy that comprised at least 10% of that stage group. For example, <10% of Stage I adenocarcinoma patients received surgery and radiation. Therefore, we did not calculate costs for this group. We modeled best supportive care for each stage/histology subgroup. All cost outputs are for monthly costs.

The linear regression equation is in the form:

Log Monthly Cost = Intercept + βAge *(Age in years) + βYear *(Calendar Year – 2000).

The “age” variable represents a patient’s age during that phase. We used age of diagnosis for the staging, surgery, and initial phase models. Since the continuing phase has the potential to span several years, we analyzed this phase using age-based sub-phases. However, for these models, one should enter a single numeric age when calculating the monthly cost. For the six-month terminal phase, we used the age of death as the age variable.

The “year” variable represents the calendar year. We used year of diagnosis as the year variable for the staging, surgery, and initial phase models. For the continuing phase models, we used the year during the age-based sub-phase and for the terminal phase models, we used year of death. Note that the year variable is scaled; for year 2000, one should enter “0”, for the year 2001, one should enter “1”, and so on.

**Table 1.** Linear regression model coefficients for the staging phase†

|  | Total Costs | | | | Patient Liability Costs | | | |
| --- | --- | --- | --- | --- | --- | --- | --- | --- |
|  | Intercept | Year | Age | Year*Age | Intercept | Year | Age | Year*Age |
| ***AJCC Stage*** |  |  |  |  |  |  |  |  |
| *Adenocarcinoma* |  |  |  |  |  |  |  |  |
| **Stage I** | NS | NS | NS | NS | NS | NS | NS | NS |
| **Stage II** |  |  |  |  |  |  |  |  |
| Best supportive care | 3.0419 | NS | 0.05787 | NS | 11.9070 | -1.2293 | -0.0706 | NS |
| Chemoradiation | 4.5701 | 0.5521 | 0.0491 | -0.0070 | NS | NS | NS | NS |
| **Stage III** |  |  |  |  |  |  |  |  |
| Best supportive care | 1.92405 | NS | 0.0783 | NS | NS | NS | NS | NS |
| **Stage IV** |  |  |  |  |  |  |  |  |
| Chemotherapy | NS | NS | NS | NS | 2.1650 | 0.9348 | 0.0644 | -0.0129 |
| *Squamous Cell Carcinoma* |  |  |  |  |  |  |  |  |
| **Stage I** |  |  |  |  |  |  |  |  |
| Best supportive care | 4.5858 | NS | 0.0504 | NS | NS | NS | NS | NS |
| Radiation | 3.5137 | NS | 0.0619 | NS | 3.2117 | NS | 0.0457 | NS |
| **Stage II** |  |  |  |  |  |  |  |  |
| Radiation | NS | NS | NS | NS | 3.4942 | 0.6661 | 0.0476 | -0.0087 |
| **Stage III** | NS | NS | NS | NS | NS | NS | NS | NS |
| **Stage IV** | NS | NS | NS | NS | NS | NS | NS | NS |
|  |  |  |  |  |  |  |  |  |
| ***Historic Stage*** |  |  |  |  |  |  |  |  |
| *Adenocarcinoma* |  |  |  |  |  |  |  |  |
| **Local** |  |  |  |  |  |  |  |  |
| Best supportive care | 6.0933 | NS | 0.0247 | NS | NS | NS | NS | NS |
| Chemoradiation | 5.9104 | 0.4767 | 0.0339 | -0.00638 | NS | NS | NS | NS |
| **Regional** |  |  |  |  |  |  |  |  |
| Best supportive care | 2.77441 | NS | 0.0653 | NS | 2.4511 | NS | 0.04876 | NS |
| Chemoradiation | 9.8192 | NS | -0.0183 | NS | NS | NS | NS | NS |
| **Distant** |  |  |  |  |  |  |  |  |
| Best supportive care | 19.0898 | -1.8694 | -0.1423 | 0.02369 | 13.9957 | -1.2133 | -0.09675 | 0.0154 |
| *Squamous Cell Carcinoma* |  |  |  |  |  |  |  |  |
| **Local** |  |  |  |  |  |  |  |  |
| Best supportive care | 4.1383 | NS | 0.0557 | NS | NS | NS | NS | NS |
| Radiation | 5.0205 | NS | 0.0440 | NS | 4.1142 | NS | 0.0348 | NS |
| **Regional** |  |  |  |  |  |  |  |  |
| Chemoradiation | NS | NS | NS | NS | 8.9375 | -0.3677 | -0.0251 | 0.0046 |
| **Distant** | NS | NS | NS | NS | NS | NS | NS | NS |

†Treatment/stage subgroups not shown if there were no significant predictors in any model or if less than 10% of patients within a stage received that treatment. For example, there were no significant predictors for the total costs, and patient liability cost models for any of the stage I EAC subgroups. NS=not significant.

**Table 2.** Linear regression model coefficients for the initial and continuing phases (AJCC Stage) †

|  | Total Costs | | | | Cancer Attributable Costs | | | | Patient Liability Costs | | | |
| --- | --- | --- | --- | --- | --- | --- | --- | --- | --- | --- | --- | --- |
|  | Intercept | Year | Age | Year*Age | Intercept | Year | Age | Year*Age | Intercept | Year | Age | Year*Age |
| ***Initial Phase*** |  |  |  |  |  |  |  |  |  |  |  |  |
| *Adenocarcinoma* |  |  |  |  |  |  |  |  |  |  |  |  |
| **Stage I** |  |  |  |  |  |  |  |  |  |  |  |  |
| Best supportive care | NS | NS | NS | NS | NS | NS | NS | NS | 7.8370 | NS | -0.0306 | NS |
| Surgery | NS | NS | NS | NS | NS | NS | NS | NS | 2.2380 | NS | 0.0420 | NS |
| Radiation | NS | NS | NS | NS | NS | NS | NS | NS | 10.9200 | -0.6479 | -0.0497 | 0.0076 |
| Chemoradiation | 10.4090 | NS | -0.0178 | NS | 10.7390 | NS | -0.0241 | NS | NS | NS | NS | NS |
| **Stage II** |  |  |  |  |  |  |  |  |  |  |  |  |
| Best supportive care | NS | NS | NS | NS | 24.3231 | -2.1611 | -0.2122 | 0.0269 | NS | NS | NS | NS |
| Chemoradiation | 10.8723 | NS | -0.0237 | NS | 11.3121 | NS | -0.0316 | NS | 8.0897 | NS | -0.0106 | NS |
| **Stage III** |  |  |  |  |  |  |  |  |  |  |  |  |
| Best supportive care | NS | NS | NS | NS | 6.4070 | 0.1703 | NS | NS | NS | NS | NS | NS |
| Chemoradiation | NS | NS | NS | NS | 10.3833 | NS | -0.0175 | NS | NS | NS | NS | NS |
| **Stage IV** |  |  |  |  |  |  |  |  |  |  |  |  |
| Best supportive care | NS | NS | NS | NS | NS | NS | NS | NS | -1.2549 | -0.2353 | 0.0981 | NS |
| Chemotherapy | 8.3414 | 0.0713 | NS | NS | NS | NS | NS | NS | NS | NS | NS | NS |
| *Squamous Cell Carcinoma* |  |  |  |  |  |  |  |  |  |  |  |  |
| **Stage I** |  |  |  |  |  |  |  |  |  |  |  |  |
| Radiation | NS | NS | NS | NS | NS | NS | NS | NS | 7.1006 | -0.0560 | NS | NS |
| Chemoradiation | NS | NS | NS | NS | 10.5698 | -0.0220 | NS | NS | 6.3243 | 0.2720 | 0.0126 | -0.0037 |
| **Stage II** |  |  |  |  |  |  |  |  |  |  |  |  |
| Best supportive care | 5.9098 | 0.2364 | NS | NS | NS | NS | NS | NS | 4.3157 | 0.1825 | NS | NS |
| Chemoradiation | 8.9261 | 0.0291 | NS | NS | 8.6950 | 0.0389 | NS | NS | NS | NS | NS | NS |
| **Stage III** | NS | NS | NS | NS | NS | NS | NS | NS | NS | NS | NS | NS |
| **Stage IV** |  |  |  |  |  |  |  |  |  |  |  |  |
| Best supportive care | NS | NS | NS | NS | -24.9726 | 8.0473 | 0.4658 | -0.1111 | NS | NS | NS | NS |
|  |  |  |  |  |  |  |  |  |  |  |  |  |
| ***Continuing Phase*** |  |  |  |  |  |  |  |  |  |  |  |  |
| *Adenocarcinoma* |  |  |  |  |  |  |  |  |  |  |  |  |
| **Stage I** |  |  |  |  |  |  |  |  |  |  |  |  |
| Surgery | 3.4369 | 0.4619 | 0.0453 | -0.006 | NS | NS | NS | NS | NS | NS | NS | NS |
| Chemoradiation | 10.5812 | NS | -0.0416 | NS | NS | NS | NS | NS | 8.0996 | NS | -0.0325 | NS |
| **Stage II** |  |  |  |  |  |  |  |  |  |  |  |  |
| Surgery | NS | NS | NS | 10.9156 | NS | -0.0566 | NS | NS | NS | NS | NS | NS |
| Chemoradiation | 9.7479 | NS | -0.0302 | NS | 10.7160 | NS | -0.0437 | NS | 7.7858 | NS | -0.0437 | NS |
| Surgery, chemo, and radiation | 0.3668 | 0.9723 | 0.0903 | -0.0133 | -6.4011 | 1.5650 | 0.1880 | -0.0219 | 1.5002 | 0.6532 | 0.0506 | -0.0088 |
| **Stage III** |  |  |  |  |  |  |  |  |  |  |  |  |
| Radiation | NS | NS | NS | NS | NS | NS | NS | NS | -15.5251 | 2.1158 | 0.3070 | -0.0314 |
| **Stage IV** |  |  |  |  |  |  |  |  |  |  |  |  |
| Chemotherapy | 20.6945 | -1.5659 | -0.1714 | 0.2096 | NS | NS | NS | NS | NS | NS | NS | NS |
| Chemoradiation | 3.6076 | 0.7730 | 0.0590 | -0.0097 | NS | NS | NS | NS | NS | NS | NS | NS |
| *Squamous Cell Carcinoma* |  |  |  |  |  |  |  |  |  |  |  |  |
| **Stage I** |  |  |  |  |  |  |  |  |  |  |  |  |
| Best supportive care | 20.5245 | -1.8937 | -0.1994 | 0.0274 | NS | NS | NS | NS | 19.1225 | -1.7006 | -0.1988 | 0.02437 |
| Chemoradiation | 6.1864 | 0.5135 | 0.0130 | -0.0065 | NS | NS | NS | NS | 7.5395 | NS | -0.0264 | NS |
| **Stage II** |  |  |  |  |  |  |  |  |  |  |  |  |
| Best supportive care | NS | NS | NS | NS | -7.7948 | 2.7442 | 0.2121 | -0.0368 | NS | NS | NS | NS |
| **Stage III** | NS | NS | NS | NS | NS | NS | NS | NS | NS | NS | NS | NS |
| **Stage IV** |  |  |  |  |  |  |  |  |  |  |  |  |
| Best supportive care | NS | NS | NS | NS | 12.4791 | -0.6990 | NS | NS | NS | NS | NS | NS |
| Chemoradiation | 11.7255 | NS | -0.0547 | NS | 11.4968 | NS | -0.0526 | NS | 0.9324 | 0.9949 | 0.0633 | NS |

†Treatment/stage subgroups not shown if there were no significant predictors in any model or if less than 10% of patients within a stage received that treatment. For example, there were no significant predictors for the total costs, cancer-attributable costs, and patient liability cost models for the stage II EAC surgery initial phase. NS=not significant.

**Table 3.** Linear regression model coefficients for the initial and continuing phases (Historic Stage) †

|  | Total Costs | | | | Cancer Attributable Costs | | | | Patient Liability Costs | | | |
| --- | --- | --- | --- | --- | --- | --- | --- | --- | --- | --- | --- | --- |
|  | Intercept | Year | Age | Year*Age | Intercept | Year | Age | Year*Age | Intercept | Year | Age | Year*Age |
| ***Initial Phase*** |  |  |  |  |  |  |  |  |  |  |  |  |
| *Adenocarcinoma* |  |  |  |  |  |  |  |  |  |  |  |  |
| **Local** |  |  |  |  |  |  |  |  |  |  |  |  |
| Best supportive care | NS | NS | NS | NS | NS | NS | NS | NS | 7.3180 | NS | -0.0249 | NS |
| Chemoradiation | 10.3900 | NS | -0.0178 | NS | 10.8080 | NS | -0.0254 | NS | 8.0720 | NS | -0.0108 | NS |
| **Regional** |  |  |  |  |  |  |  |  |  |  |  |  |
| Chemoradiation | 10.5996 | NS | -0.0190 | NS | 10.8665 | NS | -0.0243 | NS | NS | NS | NS | NS |
| **Distant** |  |  |  |  |  |  |  |  |  |  |  |  |
| Best supportive care | NS | NS | NS | NS | NS | NS | NS | NS | 0.6200 | -0.2111 | 0.0720 | NS |
| *Squamous Cell Carcinoma* |  |  |  |  |  |  |  |  |  |  |  |  |
| **Local** |  |  |  |  |  |  |  |  |  |  |  |  |
| Best supportive care | NS | NS | NS | NS | NS | NS | NS | NS | 5.0850 | 0.0827 | NS | NS |
| Radiation | NS | NS | NS | NS | 2.2040 | 0.9632 | 0.0758 | -0.0120 | 7.0500 | -0.0520 | NS | NS |
| Chemoradiation | 10.1000 | NS | -0.0139 | NS | 10.5450 | NS | -0.0219 | NS | 6.3530 | 0.2223 | 0.0116 | -0.0030 |
| **Regional** |  |  |  |  |  |  |  |  |  |  |  |  |
| Best supportive care | -1.9651 | 2.1080 | 0.1117 | -0.0253 | NS | NS | NS | NS | 4.4924 | 0.1796 | NS | NS |
| Surgery | 14.4331 | NS | -0.0948 | NS | NS | NS | NS | NS | 10.9873 | NS | -0.0745 | NS |
| Chemoradiation | 8.9823 | 0.0303 | NS | NS | 8.7622 | 0.0416 | NS | NS | NS | NS | NS | NS |
| **Distant** | NS | NS | NS | NS | NS | NS | NS | NS | NS | NS | NS | NS |
|  |  |  |  |  |  |  |  |  |  |  |  |  |
| ***Continuing Phase*** |  |  |  |  |  |  |  |  |  |  |  |  |
| *Adenocarcinoma* |  |  |  |  |  |  |  |  |  |  |  |  |
| **Local** |  |  |  |  |  |  |  |  |  |  |  |  |
| Surgery | 4.1755 | 0.4170 | 0.0351 | -0.0059 | NS | NS | NS | NS | NS | NS | NS | NS |
| Chemoradiation | 10.01620 | NS | -0.0346 | NS | NS | NS | NS | NS | 7.9211 | NS | -0.0303 | NS |
| **Regional** |  |  |  |  |  |  |  |  |  |  |  |  |
| Surgery | -3.1056 | 1.3376 | 0.1324 | -0.01763 | NS | NS | NS | NS | 0.9519 | 0.6337 | 0.0584 | -0.0087 |
| Chemoradiation | 9.1428 | NS | -0.0206 | NS | NS | NS | NS | NS | 6.9829 | NS | -0.0162 | NS |
| Surgery, chemo, and radiation | 2.8489 | 0.6902 | 0.05668 | -0.0092 | NS | NS | NS | NS | 1.5914 | 0.6450 | 0.0503 | -0.0085 |
| **Distant** |  |  |  |  |  |  |  |  |  |  |  |  |
| Chemotherapy | 22.0516 | -1.8446 | -0.0188 | 0.2451 | 23.0280 | -1.9855 | -0.2106 | 0.02719 | 20.1910 | -2.2818 | -0.1832 | 0.02938 |
| Chemoradiation | 4.0067 | 0.71846 | 0.0530 | -0.0096 | NS | NS | NS | NS | NS | NS | NS | NS |
| *Squamous Cell Carcinoma* |  |  |  |  |  |  |  |  |  |  |  |  |
| **Local** |  |  |  |  |  |  |  |  |  |  |  |  |
| Best supportive care | 20.7537 | -1.9219 | -0.2014 | 0.02762 | NS | NS | NS | NS | 19.2386 | -1.7014 | -0.2008 | 0.02443 |
| Chemoradiation | 9.2887 | NS | -0.0270 | NS | NS | NS | NS | NS | 7.5188 | NS | -0.0267 | NS |
| **Regional** |  |  |  |  |  |  |  |  |  |  |  |  |
| Chemoradiation | 9.1051 | NS | -0.0243 | NS | NS | NS | NS | NS | NS | NS | NS | NS |
| **Distant** |  |  |  |  |  |  |  |  |  |  |  |  |
| Best supportive care | NS | NS | NS | NS | 19.9408 | NS | -0.1739 | NS | NS | NS | NS | NS |
| Chemoradiation | 10.1389 | NS | -0.0345 | NS | NS | NS | NS | NS | NS | NS | NS | NS |

†Treatment/stage subgroups not shown if there were no significant predictors in any model or if less than 10% of patients within a stage received that treatment. For example, there were no significant predictors for the total costs, cancer-attributable costs, and patient liability cost models for the regional EAC surgery initial phase. NS=not significant.

**Table 4.** Linear regression model coefficients for the terminal phase†

|  | Total Costs | | | | Cancer Attributable Costs | | | | Patient Liability Costs | | | |
| --- | --- | --- | --- | --- | --- | --- | --- | --- | --- | --- | --- | --- |
|  | Intercept | Year | Age | Year*Age | Intercept | Year | Age | Year*Age | Intercept | Year | Age | Year*Age |
| ***AJCC Stage*** |  |  |  |  |  |  |  |  |  |  |  |  |
| *Adenocarcinoma* |  |  |  |  |  |  |  |  |  |  |  |  |
| **Stage I** |  |  |  |  |  |  |  |  |  |  |  |  |
| Radiation | 14.0703 | -0.6194 | -0.0629 | 0.0076 | 16.0489 | -0.7860 | -0.0929 | 0.0101 | 9.9710 | NS | -0.0423 | NS |
| **Stage II** |  |  |  |  |  |  |  |  |  |  |  |  |
| Best supportive care | -0.8503 | 1.0821 | 0.1134 | -0.0135 | NS | NS | NS | NS | -4.5750 | 1.3982 | 0.1307 | -0.0180 |
| Radiation | 14.2889 | -0.6982 | -0.0656 | 0.0084 | NS | NS | NS | NS | NS | NS | NS | NS |
| Chemoradiation | 11.1699 | NS | -0.0275 | NS | 10.6571 | 0.0355 | -0.0258 | NS | NS | NS | NS | NS |
| **Stage III** |  |  |  |  |  |  |  |  |  |  |  |  |
| Radiation | NS | NS | NS | NS | 12.6424 | NS | -0.0467 | NS | NS | NS | NS | NS |
| **Stage IV** |  |  |  |  |  |  |  |  |  |  |  |  |
| Best supportive care | 9.0717 | -0.0364 | NS | NS | NS | NS | NS | NS | NS | NS | NS | NS |
| Chemoradiation | 9.8440 | 0.0183 | -0.0114 | NS | 10.2904 | 0.0295 | -0.0204 | NS | NS | NS | NS | NS |
| *Squamous Cell Carcinoma* |  |  |  |  |  |  |  |  |  |  |  |  |
| **Stage I** |  |  |  |  |  |  |  |  |  |  |  |  |
| Best supportive care | 11.3565 | NS | -0.0307 | NS | NS | NS | NS | NS | 8.9946 | NS | -0.0342 | NS |
| **Stage II** |  |  |  |  |  |  |  |  |  |  |  |  |
| Radiation | NS | NS | NS | NS | 11.6920 | NS | -0.0350 | NS | NS | NS | NS | NS |
| Chemoradiation | 10.3890 | 0.0355 | -0.0206 | NS | 10.5390 | 0.0459 | -0.0247 | NS | 8.5140 | NS | -0.0244 | NS |
| **Stage III** |  |  |  |  |  |  |  |  |  |  |  |  |
| Radiation | 11.0856 | NS | -0.0249 | NS | NS | NS | NS | NS | NS | NS | NS | NS |
| Chemoradiation | 8.8440 | 0.0432 | NS | NS | 10.9552 | 0.0399 | -0.0298 | NS | 6.5803 | 0.0438 | NS | NS |
| **Stage IV** |  |  |  |  |  |  |  |  |  |  |  |  |
| Best supportive care | NS | NS | NS | NS | 9.2725 | -0.0608 | NS | NS | NS | NS | NS | NS |
|  |  |  |  |  |  |  |  |  |  |  |  |  |
| ***Historic Stage*** |  |  |  |  |  |  |  |  |  |  |  |  |
| *Adenocarcinoma* |  |  |  |  |  |  |  |  |  |  |  |  |
| **Local** |  |  |  |  |  |  |  |  |  |  |  |  |
| Chemoradiation | 10.3650 | NS | -0.0177 | NS | NS | NS | NS | NS | NS | NS | NS | NS |
| **Regional** |  |  |  |  |  |  |  |  |  |  |  |  |
| Chemoradiation | 8.9809 | 0.0247 | NS | NS | 8.7206 | 0.04236 | NS | NS | NS | NS | NS | NS |
| **Distant** |  |  |  |  |  |  |  |  |  |  |  |  |
| Chemoradiation | 10.0064 | 0.0170 | -0.0133 | NS | 10.3400 | 0.0217 | -0.0203 | NS | NS | NS | NS | NS |
| *Squamous Cell Carcinoma* |  |  |  |  |  |  |  |  |  |  |  |  |
| **Local** | NS | NS | NS | NS | NS | NS | NS | NS | NS | NS | NS | NS |
| **Regional** |  |  |  |  |  |  |  |  |  |  |  |  |
| Chemoradiation | 10.0761 | 0.5028 | -0.0170 | NS | 11.0252 | 0.04453 | -0.0311 | NS | 8.8535 | 0.0378 | -0.0262 | NS |
| **Distant** | NS | NS | NS | NS | NS | NS | NS | NS | NS | NS | NS | NS |

†Treatment/stage subgroups not shown if there were no significant predictors in any model or if less than 10% of patients within a stage received that treatment. For example, there were no significant predictors for the total costs, cancer-attributable costs, and patient liability cost models for any of the stage I EAC terminal phases. NS=not significant.
